# Compulsory Psychiatric Admissions in an Italian Urban Setting: Are They Actually Compliant to the Need for Treatment Criteria or Arranged for Dangerous Not Clinical Condition?

**DOI:** 10.3389/fpsyt.2018.00740

**Published:** 2019-01-08

**Authors:** Francesco Oliva, Luca Ostacoli, Elisabetta Versino, Alberto Portigliatti Pomeri, Pier Maria Furlan, Sara Carletto, Rocco Luigi Picci

**Affiliations:** ^1^Department of Clinical and Biological Sciences, University of Turin, Orbassano, Italy; ^2^Department of Neurosciences “Rita Levi Montalcini”, University of Turin, Turin, Italy

**Keywords:** involuntary hospitalization, compulsory admission, social control, need for treatment, mental health, legislation, Italy

## Abstract

**Background:** Italy was one of the first European countries adopting the need for treatment criteria for compulsory admission (CA). The aim of the present study was to confirm whether CA in an urban setting in Italy was compliant with the requested clinical criteria.

**Methods:** In this retrospective observational study, we retrieved all collected information regarding CA in Turin (Italy) from January 2006 to December 2013. All content and data reported in the CA forms, including diagnosis and clinical details, were gathered and analyzed. Comparisons between CA with and without a diagnosis of DSM-IV psychiatric disorders and between different diagnoses were performed using either parametric or non-parametric tests, depending on variable distribution.

**Results:** Three hundred and two (10.5%) of 2,870 consecutive CAs made in Turin during a lag time of 8 years were due to unknown psychiatric diagnoses (113; 3.9%) or to psychomotor agitation (189; 6.6%). The most prevalent psychiatric disorders leading to CA were schizophrenia (729; 25.4%), brief psychotic disorder (627; 21.8%), bipolar disorder episode (396; 13.8%), delusional disorder (292; 10.2%), and personality disorder (237; 8.3%). The CAs due to psychiatric disorder were longer (U = 328,875.0; *p* < 0.001) and involved patients who were more likely to be compulsorily admitted during the study period (U = 357,012.5; *p* = 0.003), to have had prior contact with a psychiatrist [$\chi_{\left(2\right)}^{2}$ = 28.34; *p* < 0.001], to have had previous admissions to a psychiatric ward [$\chi_{\left(2\right)}^{2}$ = 33.06; *p* < 0.001], to be under the care of psychiatric services [$\chi_{\left(3\right)}^{2}$ = 87.01; *p* < 0.001], and not to have concurrent alcohol [$\chi_{\left(1\right)}^{2}$ = 23.06; *p* < 0.001] and/or drug use [$\chi_{\left(1\right)}^{2}$ = 12.97; *p* < 0.001] than those due to psychomotor agitation/unspecified diagnoses.

**Conclusion:** Despite a history of 35 years of CA made according to a strict need for treatment criteria, the evaluation of CA records shows that a certain proportion of CAs appears to have been due to brief, not psychiatric, alcohol/drug related behavioral conditions. Further studies should confirm the need for law reform leading to the integration between the need for treatment and the danger criteria for CAs.

## Introduction

In Europe, Italy is one of the few countries, together with Spain, Sweden and Switzerland, in which compulsory psychiatric admission (CA) can be arranged only when the need for treatment criteria are met (1–6). Most other countries also consider certain danger criteria, with a considerable variability, from potential danger to oneself and others (e.g., France, Germany, Austria, and the Netherlands) to unacceptability for the community (Ireland and Cyprus) (1–6). As argued previously (1), the introduction of the need for treatment criteria for CA aimed to improve the psychiatrist–patient relationship with respect to community health, declining the concept of legal obligation to punish individual behavior and to protect society. According to the still-valid Italian mental health reform (Law no. 180) passed in 1978 (7), psychiatric patients have the right to be treated as well as patients with any other disorder; thus, in daily psychiatric practice, their illness should be managed using only voluntary treatments. However, in the case of particular clinical urgency, hospitalization may be implemented in a compulsory manner in order to improve treatment outcome and functional recovery. To defend, as much as possible, the need for treatment criteria, the law of establishment of the Italian National Health Service (Law no. 833) (8) imposed that a CA in Italy needs an initial clinical assessment by any medical doctor, and subsequently by a medical doctor of the Italian National Health Service, who must confirm the presence of all three of the following criteria: (a) the patient shows mental changes requiring an urgent therapeutic intervention; (b) the patient does not accept the treatment; (c) there are no conditions enabling doctors to take other timely and adequate therapeutic measures outside those achieved in a hospital. Moreover, the CA must be formally authorized by the mayor of the municipality where the patient lives and can only be undertaken in acute psychiatric wards located in public general hospitals. The maximum duration of initial involuntary placement in Italy is 7 days, one of the shortest among European countries (that ranges from a 3-day treatment period in a state of Switzerland to a 9-month one in Finland) (1–4), which can be subsequently reconfirmed for a further 7 days and so on while the criteria persist (8, 9). Lastly, it should be taken into account that CA criteria were included in the Law no. 833 in the section and clauses dedicated to the mental health (Articles 33, 34, and 35) (8). Thus, CA criteria were designed to manage only psychiatric conditions so much so that local health authorities subsequently released CA forms including a diagnosis field in order to specify the psychiatric clinical condition requiring the urgent therapeutic intervention.

Therefore, we can deduce that involuntary psychiatric admission in Italy should be related to a clinical condition that should be explicitly stated as a psychiatric diagnosis, and cannot be the consequence of behavioral manifestations that are unacceptable for the community (e.g., aggressive or hostile behavior, risk to self or others, or other dangerous behaviors).

Such early introduction and close attention to need for treatment criteria for CA in Italian mental health legislation, suggest a unique opportunity to evaluate the outcome of this health-oriented approach in patients affected by mental disorders.

The present study aimed to evaluate whether data collected during CA were informative for the actual clinical condition, according to the requested criteria provided by Italian law. As a secondary purpose, we aimed to compare patients who underwent CA due to psychiatric clinical disorders or due to other conditions, in terms of gender, age, state of birth, length of stay, comorbid alcohol/substance use disorder, previous CAs, and previous contact with mental health services.

## Materials and Methods

In this retrospective observational study, we retrieved all collected information regarding CA in Turin, Italy, from January 2006 to December 2013, by consulting the registry of the *ufficio TSO* [compulsory treatment office] of Turin, Italy. Consistent with the original CA forms, the registry record provided patient sociodemographic data (i.e., name, surname, date of birth, residence address, and responsible service) and CA details (i.e., start and end date, requested criteria, place of admission, diagnosis, and clinical observation). All data were managed in an anonymous manner, according to Local Ethical Committee compliance (notification no. 3/2016; protocol no. 001804).

To test the compliance with the Italian law concerning CA, diagnosis according to DSM-IV-TR codification was determined by searching for specific disorder keywords in the proper field of the registry record, which in turn corresponds to the same field of the CA form filled in by medical doctors during the clinical examination. The results of this search were collected according to DSM-IV-TR codification main categories (e.g., 295.xx for schizophrenia, 296.xx for bipolar disorder, etc.). To address the occurrence of a dual diagnosis, psychiatric disorders took priority over alcohol/drug use disorders in the diagnosis field, but the possible co-occurring alcohol and/or drug use were collected as two separate and dichotomous (yes/no) variables. Diagnoses that were not compliant with the DSM-IV-TR classification were collected as they were written in the diagnosis field of the original admission form.

The length of CA was calculated using start and end dates.

### Statistical Analysis

All data collections and calculations were performed using the IBM SPSS Statistics for MACOS package (Version 22.0, IBM Corporation, Armonk, NY, USA).

The annual rate of CA is expressed as the number of CAs per year per 100,000 inhabitants, using Turin population data provided by the Municipality of Turin City.

Comparison between CAs due to any DSM-IV-TR psychiatric disorder and those due to other conditions (i.e., psychomotor agitation or unspecified diagnoses) for categorical variables was performed using Pearson's χ^2^ or Fisher's exact tests, depending on the expected frequencies in each group. The adjusted residuals were calculated to allow the *post-hoc* analysis of 2 × N crosstabs. Continuous variables were evaluated using either an unpaired *t*-test or a Mann–Whitney non-parametric *U*-test, depending on whether the distribution of variables was normal or non-normal, as determined by the Shapiro–Wilk test.

Association between categorical data and each DMS-IV-TR psychiatric diagnosis was tested using Pearson's χ^2^ or Fisher's exact tests, depending on the expected frequencies in each group.

Probability tests were considered bilateral, with a type I error set at 5% (*p* = 0.05). *p*-values resulting from multiple comparisons were adjusted according to Bonferroni's correction, in order to control family error rate.

## Results

**Figure 1:**
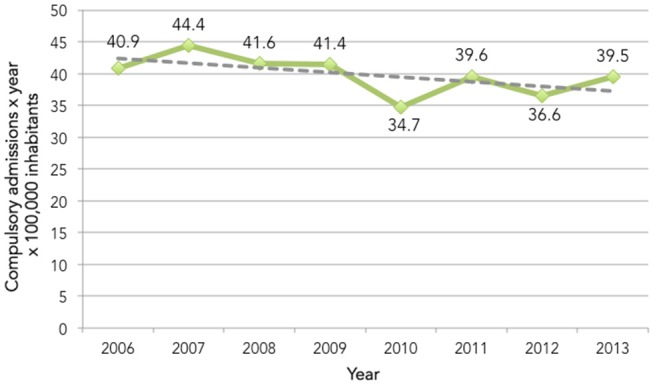
Number of compulsory admissions per year per 100,000 inhabitants in Turin.

Data from 2,870 records, corresponding to the same amount of CAs, were collected. The mean number of CAs per year per 100,000 inhabitants was 39.8 (*SD* = 3.03; Figure 1).

According to the diagnosis field content, 2568 (89.5%) CAs were due to psychiatric conditions described in the DSM-IV-TR, 189 (6.6%) were due to psychomotor agitation, and 113 (3.9%) had an empty diagnosis field, and thus were considered unspecified diagnoses.

**Figure 2:**
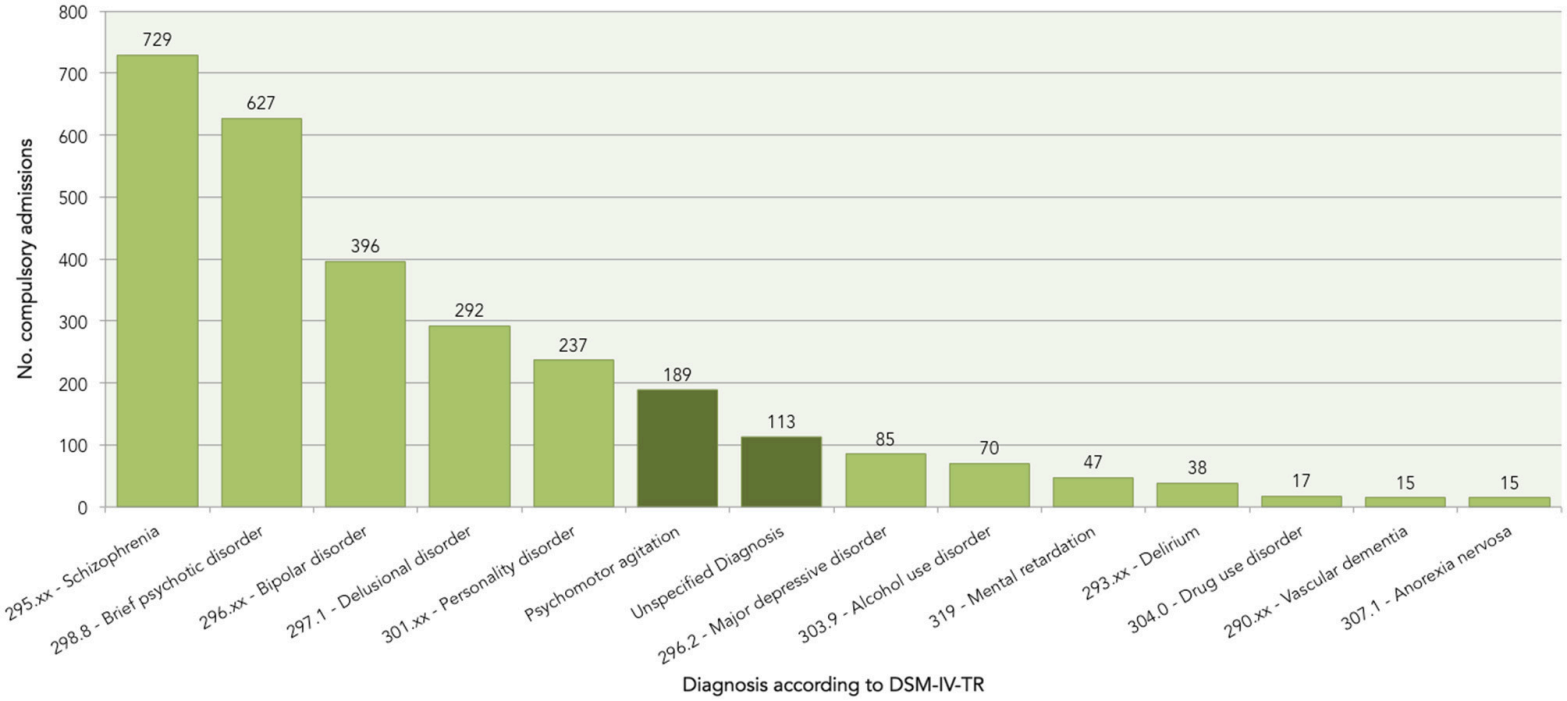
Prevalence of DSM-IV-TR psychiatric disorders and other conditions according to the diagnosis field of the compulsory admission form.

The most prevalent DSM-IV-TR psychiatric diagnoses were schizophrenia, brief psychotic disorder, bipolar disorder, delusional disorder, and personality disorder (Figure 2). These diagnoses were followed by psychomotor agitation and unspecified diagnoses, which reached a higher prevalence than the remaining DSM-IV-TR psychiatric disorders (i.e., major depressive disorder, alcohol use disorder, mental retardation, delirium, drug use disorder, vascular dementia, and anorexia nervosa).

With respect to addictive disorders, 70 (2.4%) and 17 (0.6%) were due to alcohol and drug use disorder, respectively. However, concurrent use of alcohol or drug was found in, respectively, 232 (8.1%) and 233 (8.1%) CAs.

Results of the comparison between CAs due to any DSM-IV-TR psychiatric disorder and those due to psychomotor agitation or unspecified diagnoses are reported in Table 1. CAs due to a psychiatric disorder were longer than those due to psychomotor agitation/unspecified diagnoses. Patients compulsorily admitted for psychiatric disorders were more likely to have had previous admissions to a psychiatric ward, to be compulsorily admitted during the study period, to have had prior contact with a psychiatrist, to be under the care of psychiatric services, and not to have concurrent alcohol and/or drug use than those admitted for psychomotor agitation or unspecified diagnoses.

**Table 1:** Comparison of compulsory admissions due to any DSM-IV-TR psychiatric disorder and those due to psychomotor agitation or unspecified diagnoses.

	**DSM-IV-TR psychiatric disorder**			
	**Yes 2,568 (100%), AR**	**No 302 (100%), AR**	**χ^2^ (Df)**	***p***
Gender				
F M	1,112 (43.3), 3 1,456 (56.7), −3	128 (42.4), −3 174 (57.6), 3	0.93(1)	0.761
Place of birth				
Italy Other country	2,227 (86.7), 6 341 (13.3), −6	258 (85.4), −6 44 (14.6), 6	0.39(1)	0.584
Any previous contact with a psychiatrist				
Yes No NS	1,998 (77.4), 5.3 138 (5.4), −4.5 442 (17.2), −2.3	192 (63.6), −5.3 26 (8.6), 4.5 84 (27.8), 2.3	28.34(2)	<0.001^*^
Any previous admission to a psychiatric ward				
Yes No NS	1,240 (48.3), 5.7 129 (5.0), −2 1,199 (46.7), −5.6	94 (31.1), −5.7 16 (5.3), 2 192 (63.6), 5.6	33.06(2)	<0.001^*^
Under the care of psychiatric services				
Yes CNP No NS	1,436 (55.9), 6.3 11 (0.4), −7.4 188 (7.3), −1.0 933 (36.3), −4.5	111 (36.8), −6.3 14 (4.6), 7.4 27 (8.9), 1.0 150 (49.7), 4.5	87.01(3)	<0.001^*^
Alcohol use				
Yes No	246 (9.6), −4.8 2,322 (90.4), 4.8	56 (18.5), 4.8 246 (81.5), −4.8	23.06(1)	<0.001^*^
Substance use				
Yes No	207 (8.1), −3.6 2,361 (91.9), 3.6	43 (14.2), 3.6 259 (85.8), −3.6	12.97(1)	<0.001^*^
	**Mdn (IQ)**	**Mdn (IQ)**	**U**	***p***
Age at admission	41.9 (18.8)	39.8 (19.9)	351471.0	0.008
Number of compulsory admissions during the study	1(1)	1 (0)	357012.5	0.003^*^
Length of compulsory admission	5(4)	5(4)	328875.0	<0.001^*^

*NS, Not specified; CNP, Child neuropsychiatry; AR, Adjusted residuals*.

* *Statistically significant after Bonferroni correction (p < 0.004)*.

The comparison between different diagnoses for all data collected is reported in Supplementary Table 1. CAs due to schizophrenia, bipolar disorder, and personality disorder were strongly associated with previous contact with a psychiatrist [schizophrenia, $\chi_{\left(2\right)}^{2}$ = 65.13, *p* < 0.001; bipolar disorder, $\chi_{\left(2\right)}^{2}$ = 16.17, *p* < 0.001, and personality disorder, $\chi_{\left(2\right)}^{2}$ = 19.13, *p* < 0.001], prior admission to a psychiatric ward [schizophrenia, $\chi_{\left(2\right)}^{2}$ = 28.57, *p* < 0.001; bipolar disorder, $\chi_{\left(2\right)}^{2}$ = 8.50, *p* = 0.014, and personality disorder, $\chi_{\left(2\right)}^{2}$ = 11.05, *p* = 0.004], and being under the care of psychiatric services [schizophrenia, $\chi_{\left(3\right)}^{2}$ = 153.71, *p* < 0.001; bipolar disorder, $\chi_{\left(3\right)}^{2}$ = 9.02, *p* = 0.020, and personality disorder, $\chi_{\left(3\right)}^{2}$ = 9.25, *p* = 0.031]. However, only the CAs due to personality disorder were associated with alcohol [$\chi_{\left(2\right)}^{2}$ = 52.56, *p* < 0.001] and substance use [$\chi_{\left(2\right)}^{2}$ = 76.38, *p* < 0.001]; whereas those due to schizophrenia and bipolar disorder were significantly related to the absence of these two conditions [alcohol use: schizophrenia, $\chi_{\left(1\right)}^{2}$ = 40.57, *p* < 0.001; bipolar disorder, $\chi_{\left(2\right)}^{2}$ = 5.77, *p* = 0.016 and substance use: schizophrenia, $\chi_{\left(2\right)}^{2}$ = 14.59, *p* < 0.001; bipolar disorder, $\chi_{\left(2\right)}^{2}$ = 8.97, *p* = 0.003].

Both CAs due to delirium and mental retardation were more likely to lack information regarding previous contact with a psychiatrist [delirium, $\chi_{\left(2\right)}^{2}$ = 14.76, *p* = 0.001 and mental retardation, $\chi_{\left(2\right)}^{2}$ = 12.20, *p* = 0.005], but only the former were more likely to be associated with inadequate information concerning previous admission to a psychiatric ward [$\chi_{\left(2\right)}^{2}$ = 11.95; *p* = 0.002].

CAs due to major depression disorder were significantly related to not having had previous contact with a psychiatrist [$\chi_{\left(2\right)}^{2}$ = 21.61; *p* < 0.001] and not having had prior admission to a psychiatric ward [$\chi_{\left(2\right)}^{2}$ = 9.53; *p* = 0.009]; however, these were significantly associated with alcohol use [$\chi_{\left(2\right)}^{2}$ = 6.61; *p* = 0.010].

## Discussion

According to the present study, CAs arranged in a lag of time of 8 years in the main city of northwest Italy were compliant to the Italian law though they were not always informative for the actual psychiatric condition requiring an urgent therapeutic intervention, inasmuch as more than a tenth of them was arranged for episodes of psychomotor agitation or unspecified disorders.

Contrary to other reports related to shorter and previous period of observation, the present study found a downward trend in the annual rate of CA (10–14). This might be interpreted as a real reduction of the CA rate in the last period but it should be considered with caution taking into account the main limitation of the present study, i.e., the low generalizability of results due to single urban center design.

However, it is noteworthy that a total of 302 (10.5%) CAs appear not to have been due to an actual psychiatric disorder, but likely to any mental change requiring an urgent therapeutic intervention that did not meet the DSM-IV-TR diagnostic criteria for a psychiatric disorder.

Whilst unspecified diagnosis could be explained not only by the actual lack of a psychiatric disorder diagnosis but also by other conditions (e.g., an error filling out the form, dangerous behavior not provided for by law, early stage of unclear psychiatric condition), psychomotor agitation could only be seen as the attempt to code a behavioral condition requiring urgent intervention in absence of actual psychiatric disorder. As a matter of fact, psychomotor agitation is a symptom and it is not necessarily a manifestation of a psychiatric disorder because it could occur in different non-psychiatric conditions (e.g., reactions to life events, drugs side effects, within a septic or other underlying organic disease, etc.) but if the medical doctor had been able to make a psychiatric diagnosis during the assessment he/she would not have indicated the agitation symptom only as a condition requiring urgent treatment. Moreover, as far as we concerned, a mental change requiring urgent hospitalization is likely to be covered by DSM-IV-TR classification.

Therefore, despite a specific law developed to preserve the need for treatment principle for CA including an informative proposal, acknowledged approval, and mayor of municipality authorization, some of these CAs still appear to have been due to a behavioral manifestation that was not properly related to a clinical psychiatric condition. To the best of our knowledge, no similar findings have been previously published.

Comparison of CAs due to any psychiatric disorder with those arranged for psychomotor agitation or unspecified diagnoses revealed that the latter had a higher rate of concurrent alcohol or substance use, lower rates of previous contact with psychiatric services previous admission to a psychiatric ward, and a shorter duration of involuntary hospitalization. Consistently, a recent study by Habermeyer et al. (4) reported that substance use disorder was the second most prevalent diagnosis in involuntary admitted patients in Switzerland. In line with our results, they also found that involuntary admissions due to substance use disorders had a shorter duration of stay and were more likely due to intoxication. Furthermore another study conducted in Norway by Hustoft et al. (15) recognized greater alcohol abuse and less severe psychiatric symptoms as the most important factors predicting a brief conversion from involuntary to voluntary hospitalization.

Taken together, our findings suggest that transient non-clinical conditions, often related to drug or alcohol use, may require (not necessarily) compulsory psychiatric intervention; therefore, a dedicated procedure to deal with these particular behavioral conditions should be provided in addition to compulsory psychiatric admission.

Bringing this into the need for treatment/danger criteria debate (1, 3, 16), it could be suggested that despite the extreme need for treatment orientation of the Italian psychiatric legislation (focused on psychiatrist–patient relationship and aimed at preserving the patient's state of health), certain dangerous conditions resulting from behavioral manifestation unrelated to psychiatric disorders (i.e., threat to self or others requiring an initial intervention by the law enforcement and not by the doctor) appear to require a specific intervention to be managed. This could be explained by the discrepancy between the strict need for treatment principle inspiring the early psychiatric reform proposed by Law no. 180 and the wider criteria provided by the subsequent executive Law no. 833, according to which patients with any mental change requiring urgent therapeutic intervention not applicable outside the hospital could be compulsory admitted.

Thus, the present findings highlight the need for a more comprehensive approach, similar to that adopted by the UK, Denmark, and Ireland (1–3), including the need for treatment criteria covering the more-frequent psychiatric clinical conditions and danger criteria that could be used during less-frequent but severe and dangerous behavioral manifestation, which also need to be addressed by both law enforcement and clinical intervention. Such a differentiation within the CA legislation could prevent the expected rise of patient stigmatization related to the danger criteria (17). Even more than, as recently confirmed (10), the adoption of danger criteria does not necessarily increase the rate of CA.

As regards secondary noteworthy findings, CAs due to severe psychiatric disorders such as schizophrenia, bipolar disorders and personality disorders seemed to have a higher burden on psychiatric services as patients diagnosed with these disorders had more prior hospitalizations, were more often under the care of psychiatric services and have had more previous contact with them. On the other hand, among these, personality disorders only seemed to be compulsory admitted with a concurrent use of alcohol and substances consistently with previous reports on co-occurrence between these two conditions(18, 19).

To the best of our knowledge, the present study is the first reporting that CAs due to major depressive disorder were significantly related to not having had previous contact with a psychiatrist and not having had prior admission to a psychiatric ward, suggesting that patients with this disorder are more likely to be involuntary hospitalized on the first episode. Future studies should be focused on details and conditions in which CAs due to major depressive disorder occur, taking into account also the specific severity indexes (e.g., suicidal tendencies or attempts).

Our findings are far from conclusive, especially when taking into account certain important methodological limitations. All data were concerned with the CAs of a single urban center; thus, are not representative of the entire Italian population. Moreover, these data were collected from a CA database that was not expressively designed for explicit research purposes. On the other hand, the particular context in which they come out (i.e., CA regulated by a so old and pioneering CA reform and long period of observation in a urban setting) makes them also relevant in contributing to depict the extremely heterogeneous framework of psychiatric involuntary treatment and thus in looking ahead to the drafting of a common procedure for CA able to protect right and health of both patient and community.

Therefore, our suggestion should be confirmed by further prospective observational multi-centric studies focused on CAs due to acute, non-psychiatric, alcohol-/drug-related dangerous conditions.

## Author Contributions

FO conceived and drafted the manuscript. AP collected the data. FO and EV performed the statistical analysis. RP, LO, SC, and PF participated in the design and coordination of the study. All authors read and approved the final manuscript.

### Conflict of Interest Statement

The authors declare that the research was conducted in the absence of any commercial or financial relationships that could be construed as a potential conflict of interest.
