# Supplementary material for: Compulsory Psychiatric Admissions in an Italian Urban Setting: Are They Actually Compliant to the Need for Treatment Criteria or Arranged for Dangerous Not Clinical Condition?
Source: Front Psychiatry. 2019 Jan 8;9:740. doi: 10.3389/fpsyt.2018.00740 (PMC6331583; doi:10.3389/fpsyt.2018.00740)
Supplement: Supplementary file 1 [file Table_1.DOCX]

**Supplementary Table 1. Comparison between different diagnoses for all data collected**

|  | 290.xx - Vascular dementia | 293.xx - Delirium | 295.xx - Schizophrenia | 296.xx - Bipolar disorder | 296.2 - Major depressive disorder | 297.1 - Delusional disorder | 298.8 - Brief psychotic disorder | 301.xx - Personality disorder | 303.9 - Alcohol use disorder | 304.0 - Drug use disorder | 307.1 - Anorexia nervosa | 319 - Mental retardation | Psychomotor agitation | Unspecified diagnosis | Total |  |
| --- | --- | --- | --- | --- | --- | --- | --- | --- | --- | --- | --- | --- | --- | --- | --- | --- |
|  | n (%) | n (%) | n (%) | n (%) | n (%) | n (%) | n (%) | n (%) | n (%) | n (%) | n (%) | n (%) | n (%) | n (%) | N (%) |  |
| Any previous contact with a psychiatrist  Yes  No  NS | 12 (80.0)  1 (6.7)  2 (13.3) | *  20 (52.6)  3 (7.9)  15 (39.5) | *  642 (88.1)  22 (3.0)  65 (8.9) | *  326 (82.3)  5 (1.3)  65 (16.4) | *  50 (58.8)  12 (14.1)  23 (27.1) | *  203 (69.5)  22 (7.5)  67 (22.9) | *  421 (67.1)  63 (10.0)  143 (22.8) | *  210 (88.6)  4 (1.7)  23 (9.7) | 51 (72.9)  2 (2.9)  17 (24.3) | 14 (82.4)  2 (11.8)  1 (5.9) | 11 (73.3)  0 (0.0)  4 (26.7) | *  28 (59.6)  2 (4.3)  17 (36.2) | *  117 (61.9)  17 (9.0)  55 (29.1) | *  74 (65.5)  9 (8.0)  30 (26.5) | 2179 (75.9)  164 (5.7)  527 (18.4) |  |
| Any prior admission to a psychiatric ward  Yes  No  NS | 4 (26.7)  0 (0.0)  11 (73.3) | *  8 (21.1)  2 (5.3)  28 (73.7) | *  412 (56.5)  26 (3.6)  291 (39.9) | *  206 (52.0)  9 (2.3)  181 (45.7) | *  30 (35.3)  9 (10.6)  46 (54.1) | 130 (44.5)  19 (6.5)  143 (49.0) | *  260 (41.5)  55 (8.8)  312 (49.8) | *  132 (55.7)  3 (1.3)  102 (43.0) | 25 (35.7)  3 (4.3)  42 (60.0) | 6 (35.3)  2 (11.8)  9 (52.9) | 6 (40.0)  0 (0.0)  9 (60.0) | 21 (44.7)  1 (2.1)  25 (53.2) | *  64 (33.9)  12 (6.3)  113 (59.8) | *  30 (26.5)  4 (3.5)  79 (69.9) | 1334 (46.5)  145 (5.1)  1391 (48.5) |  |
| Under the care of psychiatric services  Yes  CNP  No  NS | 4 (26.7)  0 (0.0)  2 (13.3)  9 (60.0) | 6 (15.8)  0 (0.0)  3 (7.9)  29 (76.3) | *  536 (73.5)  1 (0.1)  38 (5.2)  154 (21.1) | *  219 (55.3)  0 (0.0)  19 (4.8)  158 (39.9) | *  24 (28.2)  0 (0.0)  14 (16.5)  47 (55.3) | 150 (51.4)  0 (0.0)  28 (9.6)  114 (39.0) | *  301 (48.0)  1 (0.2)  61 (9.7)  264 (42.1) | *  141 (59.5)  5 (2.1)  14 (5.9)  77 (32.5) | 31 (44.3)  0 (0.0)  4 (5.7)  35 (50.0) | 5 (29.4)  0 (0.0)  2 (11.8)  10 (58.8) | 5 (33.3)  0 (0.0)  1 (6.7)  9 (60) | *  14 (29.8)  4 (8.5)  2 (4.3)  27 (57.4) | *  68 (36.0)  8 (4.2)  16 (8.5)  97 (51.3) | *  43 (38.1)  6 (5.3)  11 (9.7)  53 (46.9) | 1547 (53.9)  25 (0.9)  215 (7.5)  1083 (37.7) |  |
| Alcohol use disorder  Yes  No | 1 (6.7)  14 (93.3) | 7 (18.4)  31 (81.6) | *  27 (3.7)  702 (96.3) | *  25 (6.3)  371 (93.7) | *  15 (17.6)  70 (82.4) | *  13 (4.5)  279 (95.5) | *  33 (5.3)  594 (94.7) | *  54 (22.8)  183 (77.2) | *  70 (100.0)  0 (0.0) | 0 (0.0)  17 (100.0) | 2 (13.3)  13 (86.7) | 2 (4.3)  45 (95.7) | *  37 (19.6)  152 (80.4) | *  19 (16.8)  94 (83.2) | 302 (10.5)  2568 (89.5) |  |
| Substance use disorder  Yes  No | 0 (0.0)  15 (100.0) | 3 (7.9)  35 (92.1) | *  35 (4.8)  694 (95.2) | *  17 (4.3)  379 (95.7) | 8 (9.4)  77 (90.6) | 18 (6.2)  274 (93.8) | 50 (8.0)  577 (92.0) | *  54 (22.8)  183 (77.2) | 5 (7.1)  65 (92.9) | *  17 (100.0)  0 (0.0) | 0 (0.0)  15 (100.0) | *  0 (0.0)  47 (100.0) | *  30 (15.9)  159 (84.1) | 13 (11.5)  100 (88.5) | 250 (8.7)  2620 (91.3) |  |

* Statistically significant, *p* < 0.05
